# *In vivo* therapeutic efficacy of frog skin-derived peptides against *Pseudomonas aeruginosa*-induced pulmonary infection

**DOI:** 10.1038/s41598-017-08361-8

**Published:** 2017-08-17

**Authors:** Chen Chen, Maria Luisa Mangoni, Y. Peter Di

**Affiliations:** 1 Department of Environmental and Occupational Health, University of Pittsburgh, Pittsburgh, PA 15260 USA; 2 Department of Biochemical Sciences, Sapienza University of Rome, Rome, 00185 Italy

## Abstract

*Pseudomonas aeruginosa* is an opportunistic and frequently drug-resistant pulmonary pathogen especially in cystic fibrosis sufferers. Recently, the frog skin-derived antimicrobial peptide (AMP) Esc(1–21) and its diastereomer Esc(1–21)-1c were found to possess potent *in vitro* antipseudomonal activity. Here, they were first shown to preserve the barrier integrity of airway epithelial cells better than the human AMP LL-37. Furthermore, Esc(1–21)-1c was more efficacious than Esc(1–21) and LL-37 in protecting host from pulmonary bacterial infection after a single intra-tracheal instillation at a very low dosage of 0.1 mg/kg. The protection was evidenced by 2-log reduction of lung bacterial burden and was accompanied by less leukocytes recruitment and attenuated inflammatory response. In addition, the diastereomer was more efficient in reducing the systemic dissemination of bacterial cells. Importantly, in contrast to what reported for other AMPs, the peptide was administered at 2 hours after bacterial challenge to better reflect the real life infectious conditions. To the best of our knowledge, this is also the first study investigating the effect of AMPs on airway-epithelia associated genes upon administration to infected lungs. Overall, our data highly support advanced preclinical studies for the development of Esc(1–21)-1c as an efficacious therapeutic alternative against pulmonary *P*. *aeruginosa* infections.

## Introduction

Multidrug-resistant (MDR) bacterial infections represent a serious life-threat causing almost 50,000 deaths per year in Europe and in the US; and this number is expected to grow up to tenfold by 2050, killing more than cancer^1, 2^. In particular, pulmonary infections due to the Gram-negative bacterium *Pseudomonas aeruginosa* remain one of the major cause of morbidity and mortality^3^, either in intensive care units or in ventilated patients, as well as in cystic fibrosis (CF) sufferers, complicating therapy in the CF airways^4–7^. In parallel, the decrease in the pharmaceutical industry research pipeline for novel antimicrobial agents during the last three decades has resulted in an urgent need for the discovery of new strategies to address the vital problems of infectious diseases^8, 9^. Naturally occurring antimicrobial peptides (AMPs) or their derivatives stand for an appealing source for the generation of new therapeutics^9–16^. AMPs are ubiquitous in nature and act as the first line of defence against invading microorganisms^17, 18^. Although human lung epithelial cells produce AMPs (e.g. defensins and the cathelicidins LL-37)^19, 20^, most of them are present at very low concentrations and are salt-sensitive *in vitro* making it difficult for them to be active in the abnormally low pH and high-salt environment existing at the apical side of CF epithelial cells^21–23^. A promising therapeutic approach to defeat *P*. *aeruginosa* lung infections would be to exogenously apply AMPs in the lung environment. We recently identified a short-sized (21 amino acids long) derivative of the frog-skin AMP esculentin-1a, named Esculentin-1a(1–21)NH_2_ [GIFSKLAGKKIKNLLISGLKG-NH_2_, Esc(1–21)]^24, 25^, with the following attractive features: (i) a potent and rapid killing kinetics against both planktonic and biofilms forms of *P*. *aeruginosa* strains, with a pronounced membrane-perturbing activity as a plausible mode of action^26^. This is a highly non-specific mechanism which limits the induction of resistance compared to the highly selective conventional antibiotics e.g. tobramycin and ciprofloxacin that would no longer be able to recognize their specific and single target, after mutation^27^; (ii) the ability to preserve antimicrobial activity at high ionic strength, in contrast with the majority of AMPs of mammalian origin^28^; (iii) the ability to detoxify, *in vitro*, *P*. *aeruginosa* lipopolysaccharide (LPS), by inhibiting the release of TNF-α from LPS-activated macrophages^28^.

In addition, a diastereomer of Esc(1–21), named Esc(1–21)-1c, containing only two d-amino acid substitutions at the C-terminal region (dLeu^14^ and dSer^17^) was lately synthesized and found to be significantly more stable than the corresponding all-L peptide and less susceptible to enzymatic degradation (i.e. human and bacterial elastases)^29^; less toxic towards mammalian cells; more active against *P*. *aeruginosa* biofilms and more efficient in stimulating bronchial cells migration and presumably in restoring the integrity of an injured bronchial epithelium, a property which is not shown by any traditional antibiotic^28, 30^.

Even though AMPs are under investigation as novel therapeutics to defeat microbial infections, only a few studies have been reported to date on their antibacterial activity in the lungs of animal models of *P*. *aeruginosa* pneumonia^31, 32^. Remarkably, as learned from literature, when locally applied via intra-nasal or intra-tracheal route in murine models of acute lung infection, they are generally administered after a short time interval (5–15 min) from bacterial infection^33, 34^. Furthermore, to the best of our knowledge, no studies have been accomplished by the effect(s) of AMPs on the airway epithelial gene expression after *P*. *aeruginosa*-induced respiratory infection.

Taking into account the attractive properties of the two esculentin-derived peptides, we first evaluated their outcome on the bronchial epithelium permeability followed by their therapeutic efficacy in murine models of acute *Pseudomona*s lung infections, upon intra-tracheal instillation. Their effects on the expression of inflammation associated genes were also investigated. Overall, the results of our experiments have pointed out a higher *in vivo* antipseudomonal activity of the diastereomer Esc(1–21)-1c than its all-l counterpart without provoking any noticeable inflammation or damage at the lung level. Importantly, our work is the first demonstration of a frog-skin derived AMP to significantly promote lung clearance of *P*. *aeruginosa* when administered in a single dose at 2 hours after infection. The designed diastereomer demonstrated a higher antibacterial activity than the mammalian AMP LL-37, as well as a comparable potency to that of the clinically used colistin peptide^35, 36^, whose antibacterial activity is exerted through binding to LPS, the major component of the outer membrane of Gram-negative bacteria.

Hence, our data imply the diastereomer Esc(1–21)-1c as an attractive peptide for the development of future therapy for effective treatments of *Pseudomonas*-induced lung infections.

## Results

### Effect of peptides on the lung epithelial tight junction permeability

**Figure 1:**
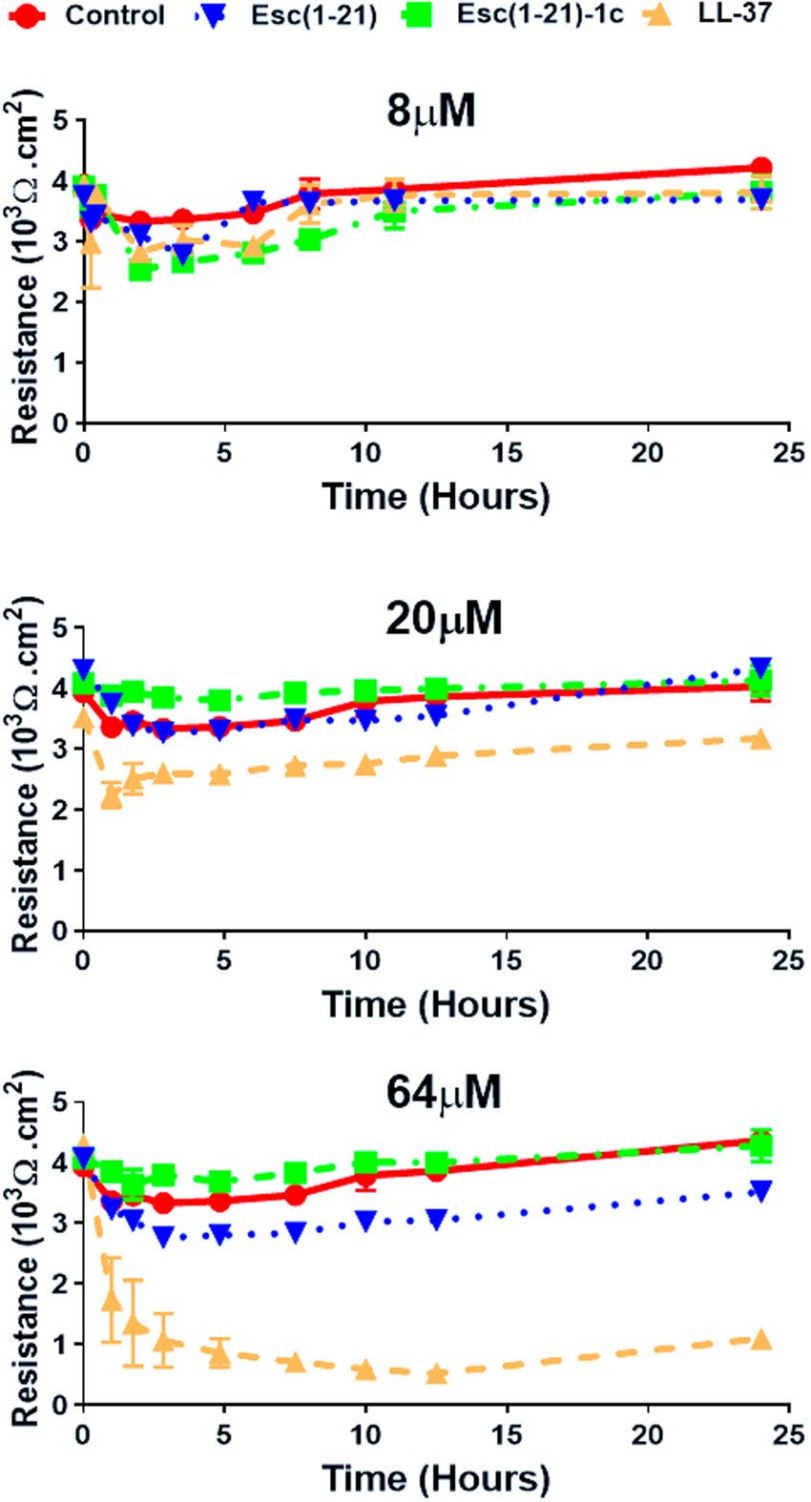
Effects of AMPs on the TEER of lung epithelial cells. Peptides in PBS were added to the apical compartment of the normal primary human bronchial epithelial cells maintained in ALI condition. TEER was measured at multiple time points (1, 2, 3, 5, 8, 10, 12 and 24 hours). Results are representative data from three independent experiments.

It was previously demonstrated that Esc(1–21) has a strong *in vitro* antipseudomonal activity, with a minimal inhibitory concentration (MIC) of 4 μM^26^. Before performing *in vivo* studies, the effect of the two esculentin derivatives on the integrity of human airway epithelium was analyzed at three selected peptide concentrations by measuring the transepithelial electrical resistance (TEER) in polarized human primary bronchial cells. Furthermore, the human cathelicidin AMP LL-37 was used as a reference. As shown in Fig. 1, when all peptides were used at a concentration corresponding to 2 × MIC (8 μM), they did not affect the epithelium integrity. When tested at a higher concentration (i.e. 20 μM), LL-37 initially decreased TEER, but this value recovered and was close to the initial one after 10 hours. Differently, when used at the higher concentration of 64 μM, it exhibited significantly detrimental effect on disturbing the epithelial cell tight junction and its negative effect persisted for a long period of time (up-triangle, broken line). In comparison, only a mild decrease in the TEER was observed for 64 μM of Esc(1–21) within 24 h (upside down triangle, broken line). Interestingly, Esc(1-21)-1c treatment had no measureable difference on TEER and did not disturb the membrane integrity at the same 64 μM concentration (square).

### Effect of peptides on the lung inflammatory response and pulmonary toxicity

Prior to analyzing the *in vivo* antipseudomonal efficacy of exogenous AMPs, the ability of the selected peptides [i.e. Esc(1–21), Esc(1–21)-1c and LL-37] to elicit host immune response and eventually lead to pulmonary toxicity upon intra-tracheal delivery, was evaluated at 24 hours after their administration. The peptides were tested at a concentration of 20 μM (2 μg/mouse) and 64 μM (7 μg/mouse).

**Figure 2:**
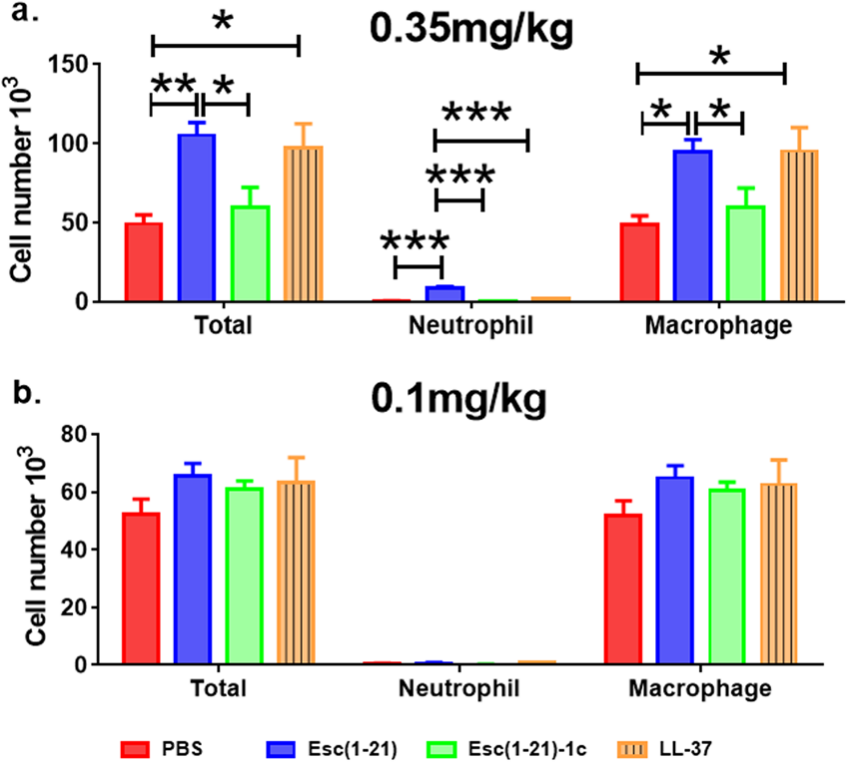
Effect of Esc(1–21), Esc(1–21)-1c and LL37 on the number of lung inflammatory cells in mice. The number of macrophages, neutrophils and total inflammatory cells in the BAL of mice was counted at 24 hours after peptide administration at 7 μg/mouse (0.35 mg/kg) (panel a) or 2 μg/mouse (0.1 mg/kg) (panel b). Results are mean ± SEM from three independent experiments; n = 4–6 mice for each group in each experiment. Following one-way analysis of variance (ANOVA), posthoc comparisons were made using the Dunnett’s multiple comparison test when the P-value was significant (*p* < 0.05). *p < 0.05, **p < 0.01, for peptide-treated mice *versus* PBS-receiving animals.

**Figure 3:**
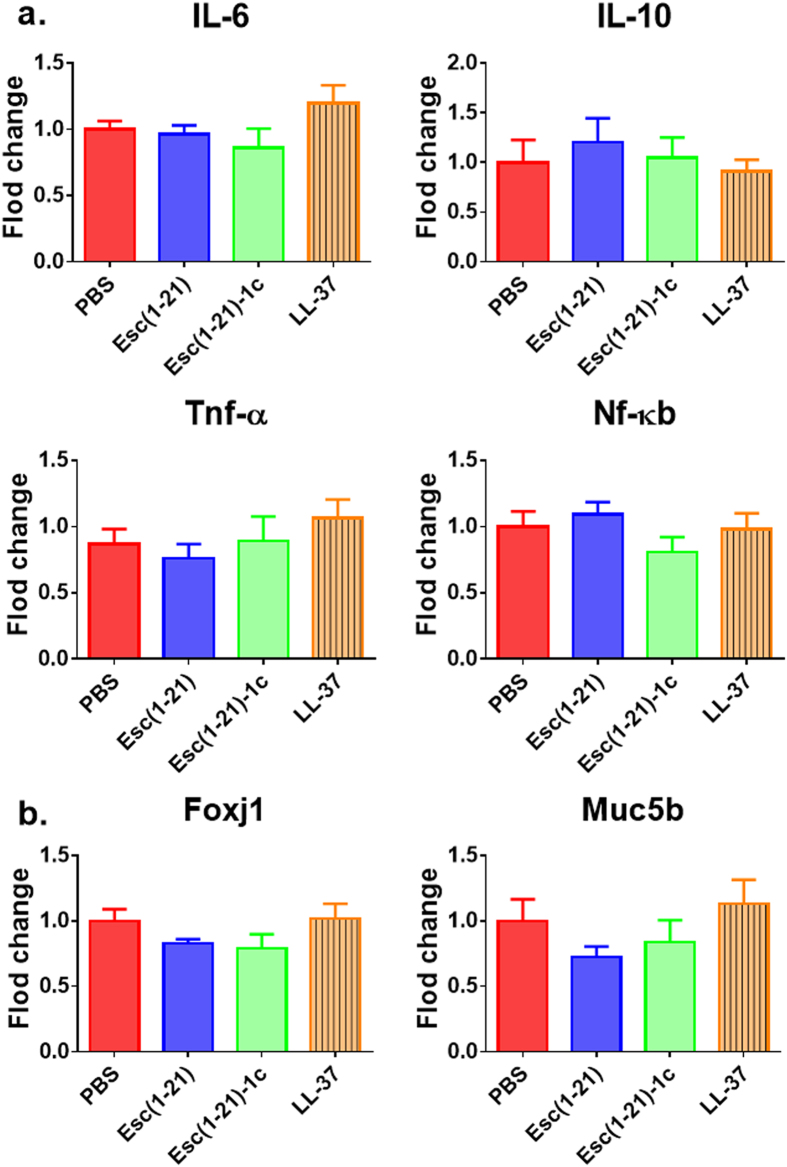
Comparison among Esc(1–21), Esc(1–21)-1c and LL-37 on the *in vivo* gene expression in the lungs of mice. Effects of peptides on the expression of different inflammatory genes (panel a) or mucociliary-associated genes (panel b) in the lungs of mice after 24 hours from peptide administration at 0.1 mg/kg. AMPs treatments at 0.1 mg/kg do not noticeably affect the expression of inflammation and mucociliary clearance-related genes. Results are mean ± SEM from two independent experiments; n = 4–6 mice for each group in each experiment.

Based on the concentrations used for determining the epithelial membrane permeability (Fig. 1), 64 μM (7 μg/mouse, 0.35 mg/kg) of both Esc(1–21) and LL-37 stimulated the host immune system, causing a considerable increase in the number of neutrophils and macrophages in the bronchoalveolar lavage (BAL) compared to the amount found in the control animals receiving the vehicle phosphate buffered saline (PBS) (Fig. 2a, red bars). However, the lung inflammatory reaction was negligible when the peptides were used at 20 μM (2 μg/mouse, 0.1 mg/kg), as demonstrated by the invariant number of total immune cells in the BAL compared to PBS-treated mice (Fig. 2b). In parallel, no observable effects on inflammation-related genes expression (such as those encoding for the cytokines IL-6, IL-10 or the tumor necrosis factor-α TNFα, and NF-kB) were observed in the lungs of mice, at 24 hours after peptide treatment at a concentration of 0.1 mg/kg (Fig. 3a).

Furthermore, no detectable pulmonary toxicity was recorded for all peptides tested. In support of this, airway epithelial cell-associated genes expression including Foxj1 (ciliated cells) and Muc5b (mucous cells), having important roles in the lung mucociliary clearance^37^, was minimally affected (Fig. 3b).

### Antimicrobial efficacy at 6 hours after bacterial infection

**Figure 4:**
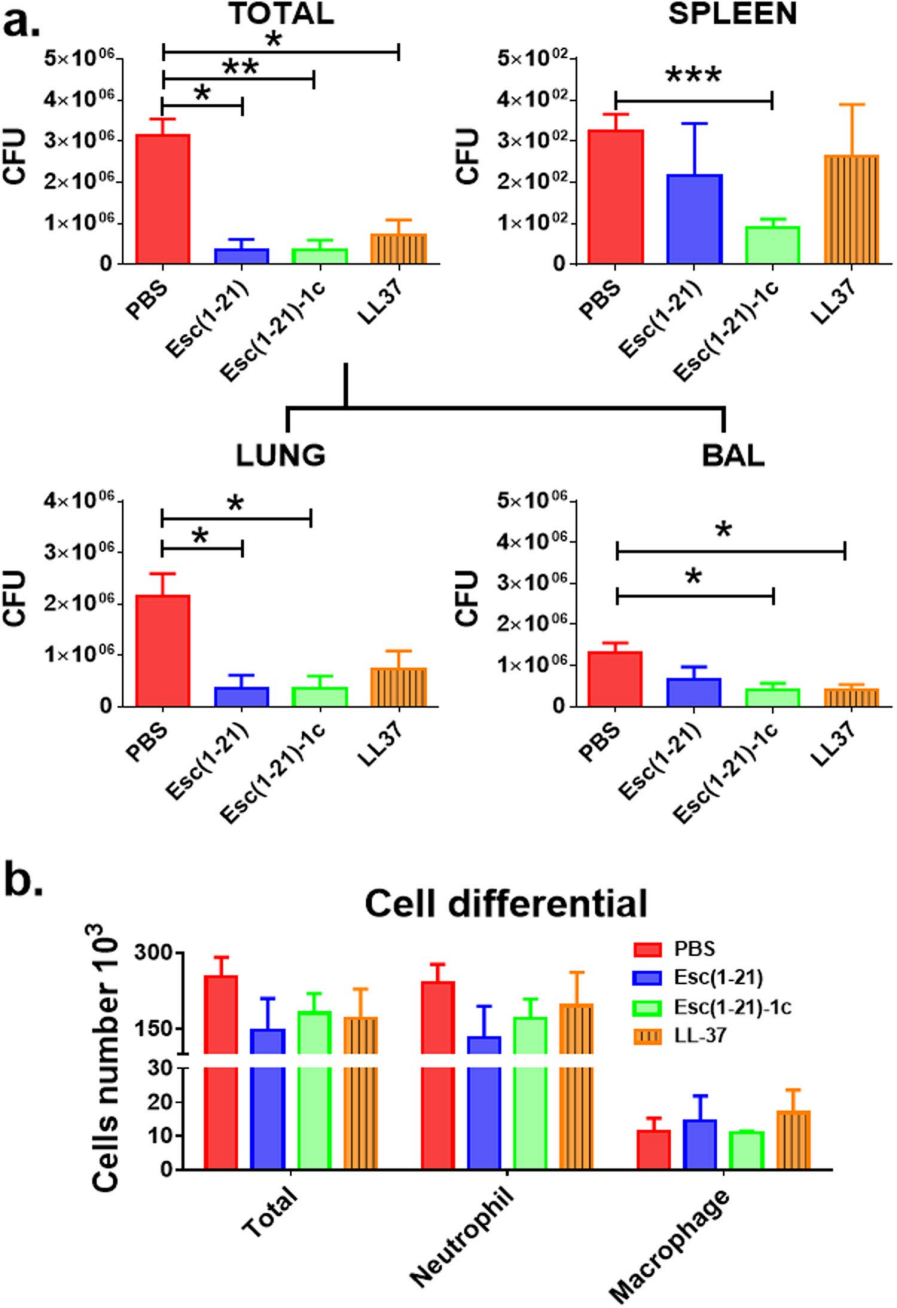
Comparison among Esc(1–21), Esc(1–21)-1c and LL-37 on the number of viable *P*. *aeruginosa* cells (CFU). CFU in mouse lungs (Total = lung homogenate + BAL) and spleens (panel a) as well as on the number of inflammatory cells in the BAL (panel b) of pulmonary-infected mice at 4 hours after peptide administration at 0.1 mg/kg were enumerated. Animals were intra-tracheally infected with 3 million PAO1 cells. The peptides were administered intra-tracheally, at 2 μg (0.1 mg/kg) at 2 hours after bacterial infection. The number of viable *Pseudomonas* cells in the lung and spleen as well as the number of inflammatory cells in the BAL were evaluated 6 hours after infection, as described in the Experimental section. Results are mean ± SEM from three independent experiments; n = 4–6 mice for each treatment group. Following one-way analysis of variance (ANOVA), posthoc comparisons were made using the Dunnett’s multiple comparison test when the P-value was significant (*p* < 0.05). *p < 0.05, **p < 0.01, ***p < 0.001 for peptide-treated animals versus PBS-treated mice.

Since no unwanted harmful action at the lung level was pointed out when all the selected AMPs were administered at 0.1 mg/kg, their *in vivo* efficacy in treating *P*. *aeruginosa*-induced respiratory infection was then examined using a mouse model of acute lung infection. The peptides were intra-tracheally administered at 2 hours after bacterial challenge and the number of colony forming units (CFUs) in lung, BAL, and spleen was determined at 6 hours after bacterial instillation. As indicated in Fig. 4a, the total lung burden as shown in number of CFU (lung homogenate + BAL) was significantly reduced (90% reduction) after AMP treatment, compared to the number found in control animals that were infected but received only the vehicle PBS (red bars). Overall, Esc(1–21)-1c revealed to be the peptide with the best *in vivo* antimicrobial efficacy, probably due to its longer biostability^29^. In addition, the significant lowering in the number of bacteria in the spleen of Esc(1–21)-1c-treated mice compared to that of control animals or those treated with Esc(1–21) or LL-37 (Fig. 4a) indicated a substantial reduction in the systemic dissemination of bacterial cells and spread of infection. In parallel, the total number of leukocytes in the infected mouse lungs after AMPs treatment was investigated. As reported in Fig. 4b, in line with the diminished bacterial burden provoked by all peptides, a lower number of immune cells, especially neutrophils, was counted in comparison with PBS-treated infected animals, 4 hours after peptide instillation (e.g. 6 hours from infection).

### Antimicrobial efficacy at 24 hours after infection with a single or double AMP administration

**Figure 5:**
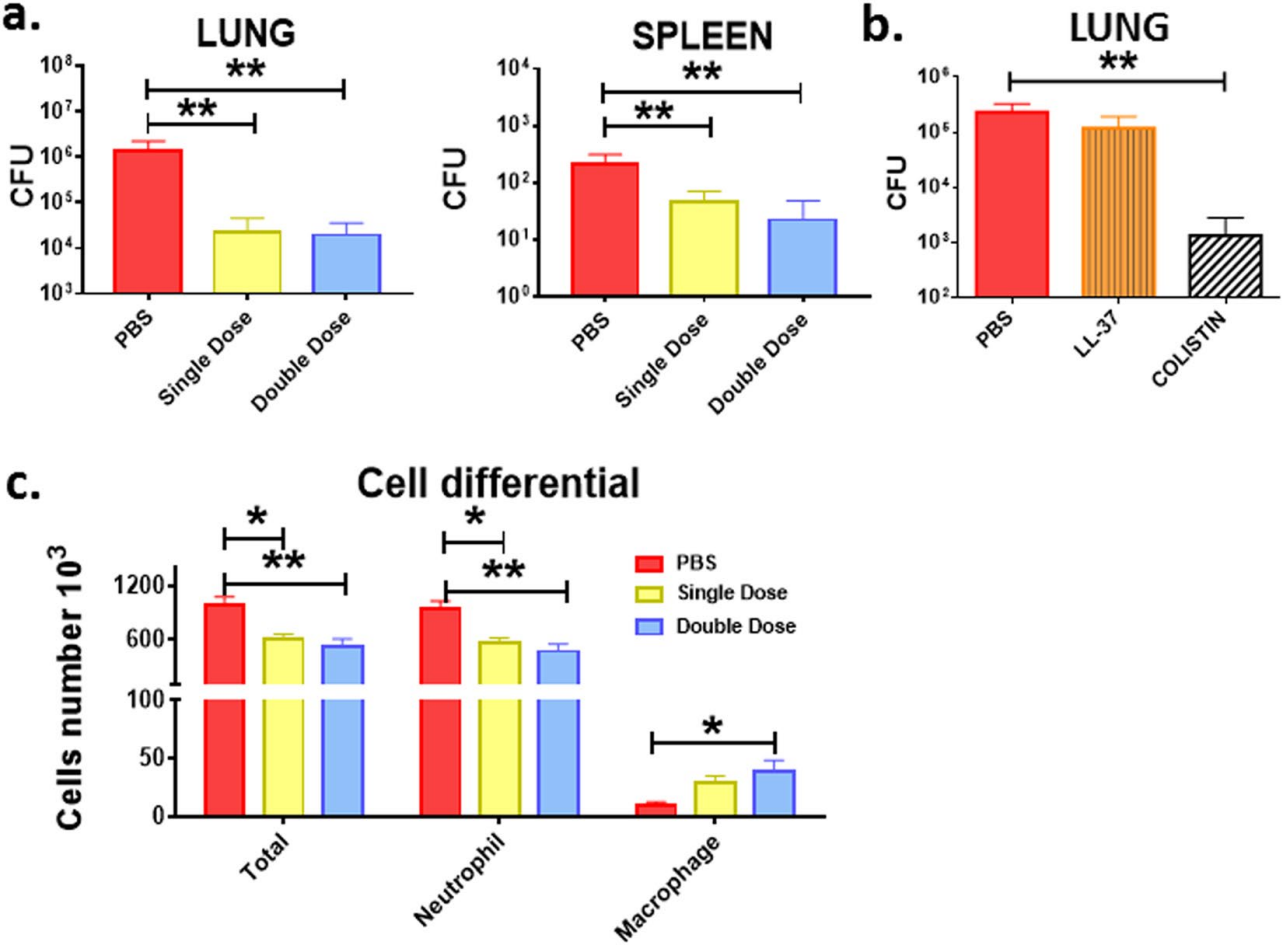
Effect of single or double administration of Esc(1–21)-1c (panel a); LL-37 and colistin (panel b) on the number of viable *P*. *aeruginosa*. The effect of single or double dosage of Esc(1–21)-1c on total leukocytes was also investigated (panel c). Bacterial burden (CFU) in the lung and spleen of infected mice was determined at 24 hours after bacterial infection. Animals were intra-tracheally infected with PAO1 cells. The peptides were instilled intra-tracheally, at 2 μg (0.1 mg/kg) at 2 hours (for single administration) and at 2 hours and 12 hours (for double administration) after bacterial infection. The numbers of viable *Pseudomonas* cells in the lung and spleen as well as the number of inflammatory cells in the BAL were counted at 24 hours after the infection, as described above. Results are mean ± SEM from three independent experiments; n = 4–6 mice for each treatment group. Following one-way analysis of variance (ANOVA), posthoc comparisons were made using the Dunnett’s multiple comparison test when the P-value was significant (*p* < 0.05). *p < 0.05, **p < 0.01 for peptide-treated animals versus PBS-treated mice.

Subsequently, the *in vivo* antipseudomonal activity of the diastereomer was further evaluated after a longer-term treatment (i.e. 24 hours from the bacterial administration). Excitingly, in this case a more noticeable antibacterial effectiveness than that from 4 hours AMP treatment was observed, with approximately 2-log reduction in the lung bacterial burden compared to the control PBS-treated infected mice (Fig. 5a). Interestingly, a second instillation of the diastereomer (0.1 mg/kg) at 12 hours after bacterial inoculation did not further enhance the therapeutic efficacy against *P*. *aeruginosa*-induced respiratory infection. Similarly, the second administration of this AMP did not further decrease the infection level of the spleen (Fig. 5a).

Importantly, the efficacy of Esc(1–21)-1c in reducing lung bacterial burden at 24 hours after Pseudomonas challenge revealed to be comparable (about 2-log reduction of lung burden) to that found for the last resort of antibiotics, i.e. colistin, in another set of experiments (Fig. 5b). In comparison, the human LL-37 almost completely lost its antimicrobial potency (Fig. 5b), presumably due to its higher susceptibility to proteolytic degradation^29^.

### Effect of Esc(1–21)-1c on the number of inflammatory cells in the BAL at 24 hours after infection

In consistence with the described results of significantly lower bacterial burden in the lungs after treatment with Esc(1–21)-1c, a substantial decrease in the total number of inflammatory cells was also recorded in the BAL of infected mice after a longer-term treatment with a single or double dosage of the diastereomer (at 0.1 mg/kg) compared to the control infected animals receiving the vehicle PBS only (Fig. 5c).

The therapeutic efficacy of the diastereomer closely correlated with the diminished number of inflammatory cells in the BAL of the animals, even though the percentage of macrophages was slightly higher than that of controls. This very low percentage of macrophages within the total leukocytes presumably reflected the lower amount of bacterial burden and the minimally recruited neutrophils to the site of infection.

### Pulmonary inflammation of infected lungs after treatment with Esc(1–21)-1c

**Figure 6:**
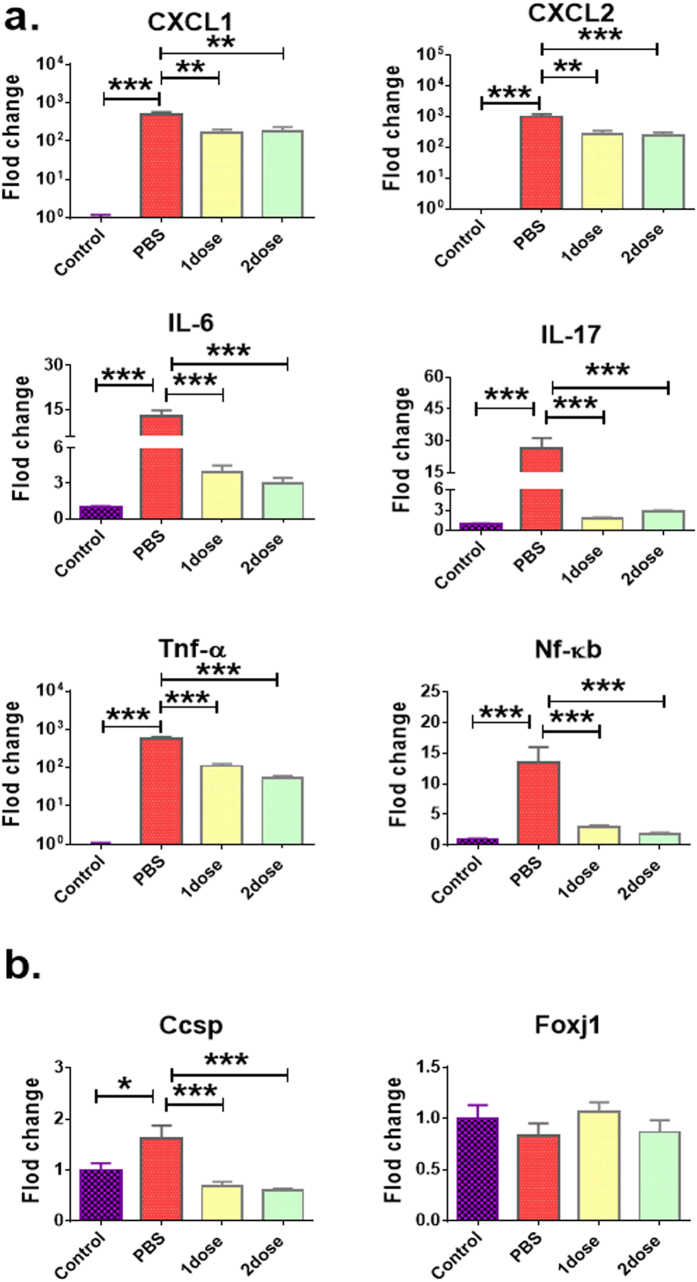
Effect of Esc(1–21)-1c on the expression level of different inflammatory-related genes (panel a) and airway epithelial-associated genes (panel b). Gene expression in the lungs of *P*. *aeruginosa* infected mice after a single or a double peptide administration (at 0.1 mg/kg) was investigated at 24 hours after bacterial infection. Mice were intra-tracheally infected with 3 million PAO1 cells. The peptides were administered intra-tracheally at 2 μg (0.1 mg/kg) at 2 hours and 12 hours after the infection. After 24 hours from bacterial challenge, the RNA was extracted from the lung tissue and quantitative PCR was performed as described^54^. Results are mean ± SEM from three independent experiments; n = 4–6 mice for each treatment group. The t-test was used to compare the means of PBS-receiving non-infected mice (control) versus PBS-treated infected animals. One-way analysis of variance (ANOVA) was used peptide-treated infected mice and PBS-treated infected animals, posthoc comparisons were made using the Dunnett’s multiple comparison test when the P-value was significant (*p* < 0.05). *p < 0.05, **p < 0.01, ***p < 0.001 for peptide-treated infected mice or PBS-receiving non-infected mice (control) versus PBS-treated infected animals.

Compared to infected mice treated with PBS, a marked reduction in the expression level of pro-inflammatory chemokines (e.g. CXCL-1, CXCL-2), cytokines (i.e. IL-6 and IL-17), and other inflammatory-related genes (Tnf-α, Nf-κb) was detected in the lungs of mice at 24 hours after treatment with a single or a double dosage of Esc(1–21)-1c at 0.1 mg/kg (Fig. 6a). Indeed, the gene-expression level was similar to that of untreated non-infected mice (control, Fig. 6a). This suggests that therapeutic treatments with the diastereomer led to a substantially attenuated inflammatory response upon *P*. *aeruginosa*-induced respiratory infection. Furthermore, bacterial infection resulted in noticeable induction of airway epithelial gene Ccsp but only mild changes in *Pseudomonas*-induced lung infection, after administration of Esc(1–21)-1c (Fig. 6b). Ccsp gene encodes for Clara/Club cell secretory protein, and serves as a marker for assessing the cellular integrity and permeability of the lung epithelium that could be induced after epithelial injury^38^. Our results clearly supported that bacterial infection-induced lung injury was significantly less after one or two dosage of Esc(1–21)-1c administration. The gene expression difference in foxj1 among various treatments was not significant.

**Figure 7:**
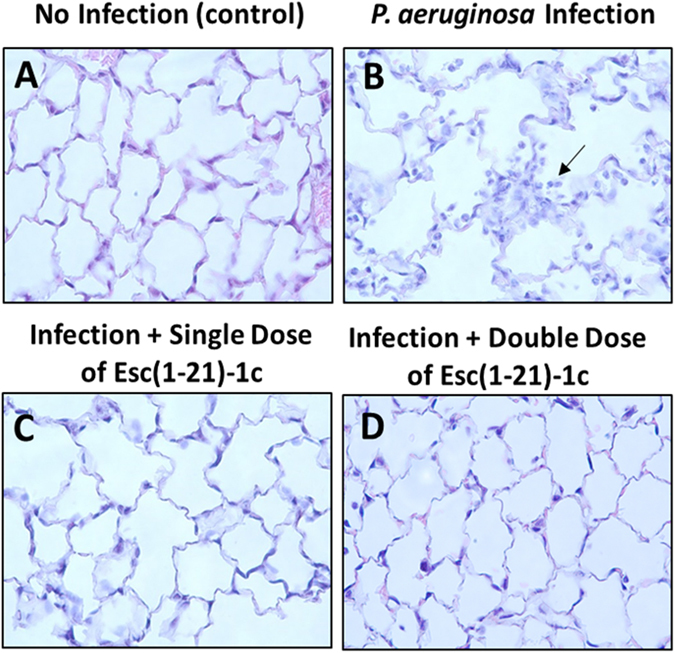
Histologic analysis of lung tissues with PAO1 challenge at 24 hours after bacterial infection. Mouse lung tissues were harvested, fixed and stained for histological evaluation without peptide treatment (**B**) or with a single (**C**) and double (**D**) administration of Esc(1–21)-1c at 0.1 mg/kg in comparison with non-infected and untreated samples. (**A**) Infected but untreated mice (**B**) exhibited more inflammation, airway lumen leukocyte accumulation (black arrow).

The efficacy of Esc(1–21)-1c in alleviating respiratory Pseudomonal infection was further assessed by mouse lung histopathological evaluation. We observed the increased number of inflammatory cells after Pseudomonas infection in both airways and alveolar regions, but the increase was more prominent in the alveolar region (Fig. 7). There was clearly more neutrophilic infiltration in the alveoli of PBS-treated mice than in AMP-treated mice at 24 hours after infection (Fig. 7).

## Discussion

Respiratory infection is the most common lung disease, especially in CF sufferers^39, 40^ and *P*. *aeruginosa* is the most predominant lung pathogen in the CF population where it drives to progressive loss of respiratory functions and shortened survival of these patients. This is mainly due to its increasing resistance to the available antibiotics and adaptation within the lung environment, favoring persistence and chronic lung colonization by *P*. *aeruginosa* ^41, 42^.

AMPs hold promise to act as lead compounds for the generation of novel therapeutic agents with fast and wide spectrum of activity, low capacity to induce resistance while displaying relevant immunomodulatory functions (e.g. LPS detoxification; wound-healing). However, a lot of AMPs loss their antimicrobial activity in biological environments and only a limited number of studies have been carried out to demonstrate the *in vivo* effect(s) of AMPs or derivatives in animal models of *P*. *aeruginosa*-induced lung infection.

We previously proved that by changing the configuration of only two amino acids in Esc(1–21), with the corresponding d-enantiomers, the resulting diastereomer Esc(1–21)-1c had a (i) significantly higher biostability, (ii) lower cytotoxicity, (iii) better antibiofilm activity, (iv) stronger ability in killing Pseudomonas internalized into bronchial cells expressing a copy of functional or mutated CF-transmembrane conductance regulator (AF508 CFTR) and (v) a more pronounced wound-healing activity in monolayers of epithelial cells^29^. All these findings contributed to make Esc(1–21)-1c a very attractive candidate for the development of new drugs against Pseudomonas-induced lung infections. Nevertheless, no evidences on its *in vivo* antimicrobial efficacy were previously reported. Here, for the first time we demonstrated a remarkable *in vivo* antipseudomonal activity of the diastereomer Esc(1–21)-1c after a single local instillation in the lungs, with a higher potency than the all-L parent peptide and the widely used and characterized human AMP LL-37. Our *in vivo* studies underlined that 4 hours peptide treatment is not an ideal time for an optimal evaluation of the *in vivo* antimicrobial effectiveness of esculentin-derived AMPs, which presumably relies on a direct bactericidal activity of the peptide. However, at this stage we cannot exclude that the respiratory epithelial cell-mediated immune modulation could also contribute to the overall host antimicrobial activity. The bacterial killing activity by Esc(1–21) and Esc(1–21)-1c would prevent inhaled/inoculated bacteria from propagate in mouse lung. Nonetheless, the bacterial infection-induced host innate immunity may be able to provide additional killing mechanism to control the severity of bacterial infection and to reduce inflammatory response. Importantly, we have found out that a single intra-tracheal instillation of Esc(1–21)-1c, at a very low peptide dosage i.e. 20 μM (0.1 mg/kg), is sufficient to cause approximately 2-log reduction in the lung Pseudomonas burden, within 24 hours from bacterial challenge. The data suggest that the diastereomer Esc(1–21)-1c likely has a sustained residence time in the lungs, due to its higher biostability and resistance to proteolytic degradation than Esc(1–21) and LL-37, according to our previously published data^28, 29^. Thus, it would continue to display microbicidal effects at longer-term. In comparison, the *in vivo* antipseudomonal activity of Esc(1–21)-1c resulted to be similar in bacterial reduction to that of colistin, which is extensively used in clinical practice^43^. Note however that colistin is a peptide which is active only against Gram-negative bacteria and it easily induces microbial resistance^31, 44, 45^, most likely because of modifications to the phosphate groups of lipid A and core oligosaccharide moieties of LPS, weakening colistin binding to it. Yet, colistin resistance in humans without prior exposure to this peptide has been recently disclosed; and this is an important concern to public health^46^.

Differently, the *in vivo* antibacterial efficacy of LL-37 was mostly lost at longer-term (Fig. 5b), probably due to its higher susceptibility to proteases^29^. This is in contrast with previous findings by Beagumont and colleagues showing that in a murine model of acute *P*. *aeruginosa* lung infection, LL-37 enhanced bacterial clearance *in vivo* only over 24 hours, by means of neutrophils recruitment, while no effect was obtained 6 hours after infection^47^. However, in their work, bacteria and peptide were co-administered via intranasal instillation rather than intra-tracheally. This latter administration route provides a more direct introduction of bacteria into the lungs and a more reproducible infection that can be more representative of pneumonia, generally leading to establishment of infection in the lower regions of the lungs^48^. In addition, intra-tracheal administration of a drug better mimics an aerosol-based therapy. Furthermore, immediate administration of LL-37 following bacterial inoculation as used in the paper^47^ did not allow sufficient time for the bacteria to adapt to the lung microenvironment before being exposed to LL-37. Note that this previously used experimental procedure could not provide a valuable indication of the antimicrobial effectiveness of AMPs *in vivo*. Thus, in this study, we purposely delayed the AMP administration at 2 hours after bacterial infection to better reflect the real life infectious conditions.

Finally in our hands, LL-37 negatively disturbed bronchial epithelial cell tight junction (Fig. 1). This could potentially result in elevated cytotoxicity to the lung epithelial cells and limit its practical use in exogenously supplying viable therapy for lung bacterial infection.

To the best of our knowledge, no indication on the effect of AMPs on the expression of airway epithelia-associated genes upon their administration to infected or non-infected lungs has been provided so far. This is a fundamental issue that deserves to be investigated in order to explore the clinical safety of a new drug. Indeed, besides experiencing the AMPs effect on the bacterial clearance, it is also meaningful to get insight into their potential beneficial/toxic effect on the lung epithelium, *in vivo*.

Our findings have emphasized that two amino acids substitution in Esc(1–21) with the corresponding d-enantiomers can reduce the peptide’s cytotoxicity and withstand the feasibility of using the diasteremer to alleviate pneumonia severity. Furthermore, we have also shown that in addition to having a therapeutic outcome, the diastereomer does not induce any undesirable side effect at the lung, but it is able to abate the inflammatory response upon bacterial infection, as corroborated by the results of immune cell differential counts, gene expression and lung histology analysis.

In summary, the results of our work suggest a strong therapeutic potential of diastereomer Esc(1–21)-1c in treating bacterial infection and concur to support further advanced preclinical studies aimed at developing it as a viable lead compound for the manufacture of new peptide-based formulation(s) for topical treatment of Pseudomonas lung infection.

## Methods

### Materials and chemicals

Synthetic Esc(1–21) and its diastereomer Esc(1–21)-1c (Table 1) were purchased from Chematek Spa (Milan, Italy). Briefly, each peptide was assembled by step-wise solid-phase synthesis using a standard F-moc strategy and purified via RP-HPLC to a purity of 98%, while the molecular mass was verified by mass spectrometry. Colistin sulphate was purchased from Sigma (St. Louis, MO) and the natural AMP human cathelicidin LL-37 (Table 1) was synthesized by Genscript (Piscataway, NJ). BronchialLife Epithelial Airway Medium was purchased from Lifeline Technology (Frederick, MD). B-ALI Bronchial Air Liquid Interface BulletKit was purchased from Lonza (Walkersville, MD). Eagle’s minimum essential medium (EMEM), was purchased from Sigma (St. Louis, MO), and tryptic soy broth (TSB) and trypitc soy agar (TSA) were purchased from MP Biomedicals (Santa Ana, CA).

**Table 1:** Primary structure of the peptides under study.

Peptide	Primary structure^a^
Esc(1–21)	GIFSKLAGKKIKNLLISGLKG-NH_2_
Esc(1–21)-1c	GIFSKLAGKKIKN***L***LI***S***GLKG-NH_2_
LL-37	LLGDFFRKSKEKIGKEFKRIVQRIKDFLRNLVPRTES

^a^ d-amino acids are in italics and bolded.

### Bacteria

The *Pseudomonas aeruginosa strain* (PAO1, ATCC BAA-47) was used for all the experiments. For each experiment, an aliquot of bacteria was grown overnight at 37 °C in TSB with shaking at 225 rpm to achieve a stationary phase suspension. Afterwards, an aliquot was diluted 1:5 into fresh TSB and incubated at 37 °C for additional 2 h at 37 °C to reach an exponential growth phase. Bacterial cells were harvested by centrifugation at 1,500 × g for 10 min, washed twice and resuspended in PBS to adjust the concentration at 6 × 10^7^ CFU/ml, for use in the experiments.

### Primary bronchial epithelial cells

Fully differentiated primary human bronchial epithelial (HBE) cell cultures were derived from lungs removed at the time of lung transplantation from the Center for Organ Recovery and Education. Cells were prepared using previously described methods approved by the University of Pittsburgh IRB^49, 50^. Briefly, bronchi from the 2nd to 6th generations were collected, rinsed, and incubated overnight in EMEM at 4 °C. The bronchi were then digested in MEM containing protease XIV and DNase (0.2%). The epithelial cells were removed and collected by centrifugation and then resuspended in BronchiaLife medium and plated onto collagen-treated tissue culture flasks. When 80–90% confluence was reached, the passage 0 cells were trypsinized and seeded onto collagen-coated transwell permeable supports (Corning #3450, 10^5^ cells/well). B-ALI Bronchial Air Liquid Interface Medium was replaced three times a week on both apical and basolateal sides of the permeable supports up to 8–10 days. Subsequently the apical medium was removed and the cultures were maintained at air liquid interface (ALI) to promote a further polarization and differentiation of the epithelium. The basolateral medium was changed twice weekly.

### Animals

Wild-type C57BL/6J female mice were purchased from Jackson Laboratory (Bar Harbor, ME) and maintained in a specific pathogen-free status in a 12 hours light/dark cycle. All procedures were conducted using mice 6–8 weeks of age maintained in ventilated microisolator cages housed in an American Association for Accreditation of Laboratory Animal Care (AAALAC) approved animal housing facility. Protocols and studies involving animals were conducted in accordance with National Institutes of Health guidelines and approved by the Institutional Animal Care and Use Committee (IACUC) at the University of Pittsburgh.

### Lung epithelium permeability

Lung epithelial integrity in polarized primary bronchial cells was examined by measuring the TEER using an electronic resistance system (Millicell ERS-2, EMD Millipore). *Ex vivo*-differentiated lung epithelium was grown on ALI in 24-transwell plates (Corning, Tewksbury, MA) using BronchiaLife Epithelial Airway Medium in the basolateral compartment. Peptides dissolved in PBS at different concentrations were added onto the apical surface of the differentiated epithelial cells in a final volume of 100 μl. A sterile electrode was applied onto the apical side of the transwell insert containing the cultured epithelial cells with/without peptide treatments and the electrical resistance was measured three times for comparison at different time intervals.

### Bronchoalveolar lavage and differential cell counts

Mice were anesthetized with inhalation of isoflurane and the peptide was intra-tracheally administered in 50 µl of PBS at 20 μM and 64 μM corresponding to a peptide dosage of 0.1 mg/kg and 0.35 mg/kg, respectively. Control mice were instilled with 50 µl of PBS without peptide. After 24 hours of treatment, mice were anesthetized with 2.5% tribromoethanol (Avertin). The trachea was cannulated, the lungs were lavaged twice using 1 ml saline, and the BAL samples collected as previously described^51^. The number of live immune cells in the BAL was determined by a Vision Cell Analyzer automatic cell counter (Nexcelom, Lawrence, MA). An additional aliquot was placed onto glass microscope slides (Shanon Cytospin; Thermo Fisher, Pittsburgh, PA), stained with Diff-Quick (Thermo Fisher Scientific, Waltham, MA); cell differential was determined microscopically. A total of 400 cells of every slide were counted at least twice independently for inflammatory cell differential counts. In other sets of experiments, cell differential counts were evaluated in the BAL of infected mice treated with or without peptides at 6 hours or 24 hours after peptide administration, as indicated.

### Murine infection model

Mice were anesthetized with inhalation of isoflurane and instilled with PAO1 bacteria (sensitive to all peptides), through intra-tracheal inoculation of ~3 × 10^6^ CFU per mouse in 50 µl PBS. Two hours after the bacterial infection, the peptide was intra-tracheally administered at desired dosage in 50 µl of PBS. Control mice were instilled with 50 µl of PBS without peptide. For repeated dosage experiments, the peptide was intra-tracheally instilled at 2 hours and 12 hours after bacterial infection.

### Determination of bacterial burden

After 6 hours or 24 hours of bacterial instillation, the numbers of CFU in lungs, BAL and spleen were determined by serial dilution on TSB agar plates. Mice (5–6 mice/group) were anesthetized with 2.5% tribromoethanol. The trachea was cannulated, the lungs were lavaged twice using 1 ml saline, and the BAL samples collected. The left lung lobe was homogenized in 1 ml saline and placed on ice. Dilution of 100 μl of lung tissue homogenate or BAL was mixed with 900 μl PBS. Four serial 10-fold dilutions in saline were prepared and plated on TSB agar plates and incubated overnight at 37 °C, each dilution plated in triplicate, for counting.

### Gene expression analysis by quantitative real-time PCR

The peptides’ effect on the expression of different inflammatory genes or airway epithelial cells-related genes after peptide administration at 0.1 mg/kg to non-infected or infected mice was determined, as indicated. Briefly, total mRNA was isolated from the right lung tissues using Trizol reagent. Quantitative PCR was performed using ABI 7900HT (Applied Biosystems, Foster City, CA) and primers of all genes tested including IL-6, IL-10, Tnf-α, Nf-κb, Muc5b, Foxj1. Validation tests were performed to confirm equivalent PCR efficiencies for the target genes. Test and calibrator lung RNAs were reverse transcribed using a high-capacity cDNA reverse transcription kit (Life Technologies) and PCR was amplified as follows: 50 °C for 2 min, 95 °C for 10 min, 40 cycles; 95 °C for 15 s; 60 °C for 1 min. Three replicates were used to calculate the average cycle threshold for the transcript of interest and for a transcript for normalization (β-glucuronidase; Assays on Demand; Applied Biosystems). Relative mRNA abundance was calculated using the ΔΔ cycle threshold (Ct) method.

### Lung histopathology

Lung tissues were harvested from mice at 24 hours after infection and compared with those without infection or after infection and peptide treatment (single or double administration at 0.1 mg/kg, as described above). Afterwards, they were fixed *in situ* with 4% paraformaldehyde for 10 minutes with the chest cavity open. The right lobe was embedded in paraffin and 5 μm sections were prepared. Sections were stained with hematoxylin and eosin, and histological evaluation was performed to examine bacterial infection-induced pathological severity. The stained lung sections were evaluated in a double-blind fashion under a light microscope, as described previously^52, 53^.

### Data analysis

Data are expressed as mean ± SEM. Statistical comparisons between groups of mice were made using ANOVA, followed by Dunnett’s multiple comparison test or Tukey–Kramer test (one way ANOVA). A *p* value < 0.05 was considered to be statistically significant.

### Data Availability

The datasets generated during and/or analyzed during the current study are available from the corresponding author on reasonable request.
